# Asymmetric reactions in continuous flow

**DOI:** 10.3762/bjoc.5.19

**Published:** 2009-04-29

**Authors:** Xiao Yin Mak, Paola Laurino, Peter H Seeberger

**Affiliations:** 1 Department of Biomolecular Systems, Max Planck Institute of Colloids and Interfaces, Research Campus Golm, D-14424 Potsdam, Germany

**Keywords:** asymmetric catalysis, biocatalysis, continuous flow, microreactors, solid phase synthesis

## Abstract

An overview of asymmetric synthesis in continuous flow and microreactors is presented in this review. Applications of homogeneous and heterogeneous asymmetric catalysis as well as biocatalysis in flow are discussed.

## Introduction

While many technological advancements have been made over the years in order to facilitate the day-to-day endeavors of the laboratory chemist, an aspect that has been reconsidered only recently is the *format* in which organic transformations are carried out. Continuous (micro)flow systems are gaining recognition as good, and in some cases, even better alternatives to the traditional ‘round-bottomed flask’ concept, which is still typically widely used for most chemical reactions [1–8]. Many advantages have been attributed to the use of flow devices, such as improved heat and mass transfer as well as mixing, and also easier scale-up and reproducibility due to the precise control over reaction conditions in these devices. Continuous flow technology has excellent potential for the integration of a high level of automation and for the incorporation of on-demand reaction analysis. This can be advantageous for applications such as high-throughput screening and synthesis, as well as for the continuous production of significant quantities of compound at higher efficiency and lower costs [1–8].

Stereoselective transformations are among the many different classes of reactions that have been investigated using continuous flow technology. The development of new methods for the synthesis of enantiopure building blocks and intermediates is important. Asymmetric catalysis, in particular, has come to the forefront as a highly economical and efficient means for the generation of chiral compounds, whereby achiral starting materials are transformed directly into enantioenriched products using only minute amounts of a renewable chiral component [9]. The potential applications of continuous flow technology as a valuable tool for asymmetric synthesis has been demonstrated in terms of fast optimization studies, improved control of reaction conditions and in the case of heterogeneous catalysis, potential long term use of the catalysts. This review will highlight some of the continuous flow asymmetric reactions described to date.

## Review

### Homogeneous catalysis

**Scheme 1:**
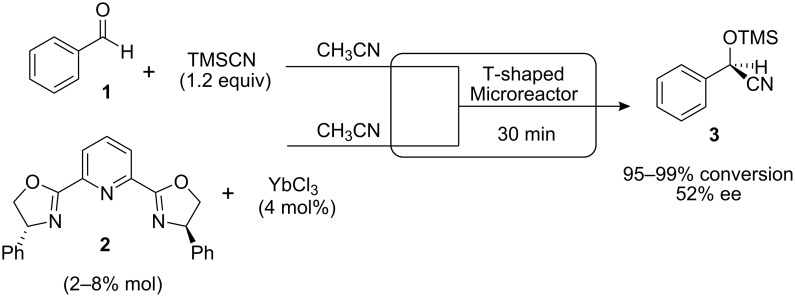
Enantioselective addition of trimethylsilyl cyanide to benzaldehyde.

Only a few examples of homogeneous enantioselective reactions in continuous flow have been reported, and these have been performed in microfluidic flow systems [10–11] consisting usually of micro-scale channels. The use of microreactors has been found to be especially useful for catalyst/ligand screening, as only low loading of reagents is required. The enantioselective silyl-cyanation of benzaldehyde (**1**) catalyzed by lanthanide(III)-PyBox complexes was investigated using a T-shaped borosilicate microreactor and electroosmotic flow (Scheme 1) [12]. The reaction was initially screened with different lanthanide (III) complexes such as Ce(III), Yb(III) and Lu(III). Further efforts were focused on screening the effect of diverse additives and also the applied voltages, in order to maximize the enantioselectivity and conversion of the reaction. While enantioselectivities for formation of cyanohydrin **3** were found to be comparable to analogous batch reactions, reactivity was observed to be higher in the microreactor.

**Scheme 2:**
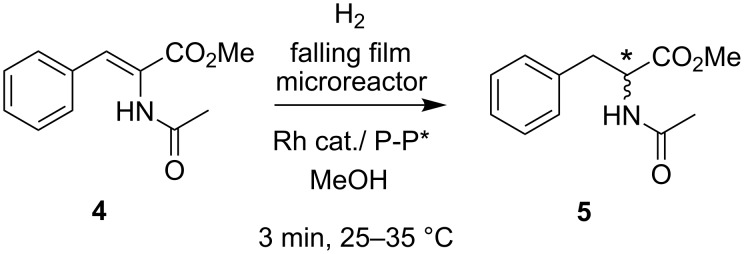
Asymmetric catalytic hydrogenation in a falling-film microreactor.

A single-channel, falling film microreactor designed specifically for efficient gas-liquid phase contact was used to screen the asymmetric hydrogenation of (*Z*)-methyl acetamidocinnamate **4** and related substrates (Scheme 2) [13–14]. Seventeen chiral phosphines were screened for reactivity and enantioselectivity with the rhodium catalyst [Rh(COD)_2_]BF_4_ within a 3 min residence time. With this device, very low catalyst/ligand loadings were used per run (ca. 0.1 μg of Rh catalyst), providing reliably reproducible results. Reactivity in the microreactor was found to be significantly higher when compared to a carousel-type reactor typically used for parallel synthesis.

**Scheme 3:**
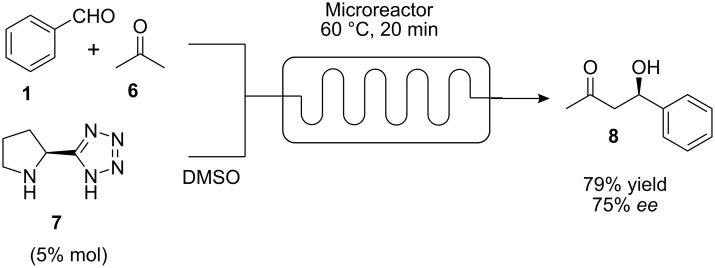
Aldol reaction catalyzed by 5-(pyrrolidine-2-yl)tetrazole.

Organocatalytic asymmetric aldol reactions in microflow devices were recently reported by our laboratory [15]. The aldol condensation of various aromatic aldehydes with acetone was carried out at higher temperatures than previously reported in batch, resulting in shorter reaction times and lower loadings of the organocatalyst **7** (Scheme 3). Slightly higher yields and selectivities, compared to reactions in both batch and in the microwave were obtained. This study was extended to an example using cyclohexanone as donor, and subsequently to a Mannich reaction with α-iminoglyoxylate.

### Supported and heterogeneous catalysis

Chiral catalysts and ligands are often expensive and the complete separation of these components from reaction products can frequently be quite challenging. Degradation products and leaching (in the case of transition metal-catalyzed reactions) also can be problematic in large-scale syntheses, especially in pharmaceutical applications. Consequently, reactions based on the use of supported catalysts are in many ways a more attractive option for the stereoselective synthesis of chiral compounds. Supported catalysts are in principle recoverable and, ideally, recyclable. With potential longer term usage, chemical processes can become more economically and environmentally friendly [16–17].

In conjunction with continuous flow, the reaction and separation of catalysts can be performed simultaneously. The mechanical degradation of the support material can lead to significantly shortened lifetimes of the supported reagent. In flow no mechanical stirring or agitation is required, thus avoiding this problem, and this can lead to higher overall productivity. However, one of the main difficulties of asymmetric synthesis using solid-supported catalysts is the development of an immobilization strategy that maintains both good stereoselectivity and catalyst activity. Selectivities obtained using homogeneous catalysts that work well in solution phase can often be significantly reduced when heterogenized [18]. Soluble supported catalysts have also been developed, particularly in form of dendrimers that can be separated from the reaction mixture via membrane filtration, a process that is also applicable in continuous flow [19–22]. This following section summarizes some recent developments in continuous flow asymmetric reactions using immobilized catalyst systems.

**Scheme 4:**
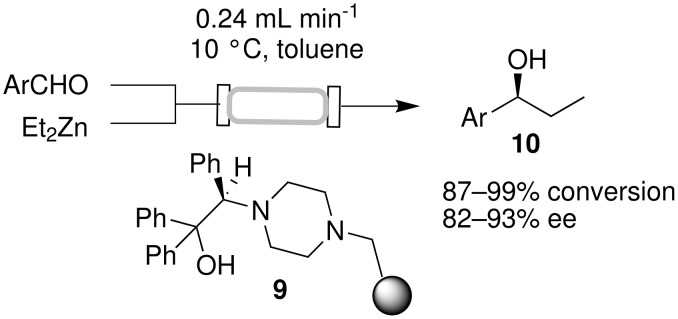
Enantioselective addition of diethylzinc to aryl aldehydes.

An asymmetric reaction that has been examined extensively in continuous flow using supported catalysts is the enantioselective addition of diethylzinc to benzaldehyde [23–28]. A fast, single pass continuous flow process for the addition of diethylzinc to various aldehydes was described recently (Scheme 4) [27]. Reagent solutions were fed by piston-pump driven flow through a fritted column packed with chiral amino-alcohol functionalized Merrifield resin **9**. Under optimal conditions (10 °C, a flow rate of 0.24 mL min^−1^ and a slight excess of Et_2_Zn) a residence time of only 9.8 min was required for 98% conversion of benzaldehyde to the desired (*S*)-1-phenyl-propanol, with 93% enantioselectivity. In comparison, the reaction time in batch for the analogous reaction was 1.5 h. Gram quantities (2.61 g) of (*S*)-1-phenyl-propanol was produced within 3 h, at a through-put of 4.4 mmol/h per gram of resin. The chiral resin was used for 6 h for three consecutive reactions using different aldehyde substrates; identical conversions and enantioselecitivities as for individual runs were observed.

**Scheme 5:**
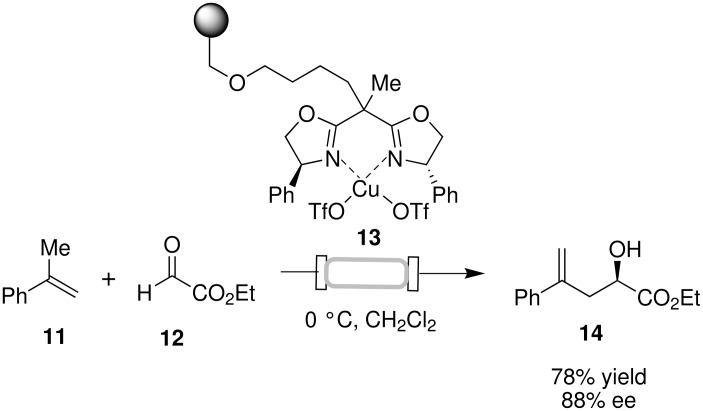
Glyoxylate-ene reaction in flow.

Another asymmetric C–C bond forming reaction that has been studied in flow is the PyBox-metal complex-catalyzed carbonyl ene reaction [29]. Salvadori and co-workers described the use of a flow reactor comprised of a stainless steel column packed with PyBox ligand functionalized polystyrene **13** for the ene reaction of ethyl glyoxylate with α-methylstyrene (Scheme 5). At a flow rate of 0.015–0.025 mL min^−1^, an 83% conversion to the desired product **14** was achieved. After initial loading of the Cu(OTf)_2_ catalyst, the column was used for up to five runs (over 80 h), with essentially no erosion of enantioselectivity and yield observed between each run and without the need for catalyst regeneration, providing 78% total yield of **14** with 88% ee.

**Scheme 6:**
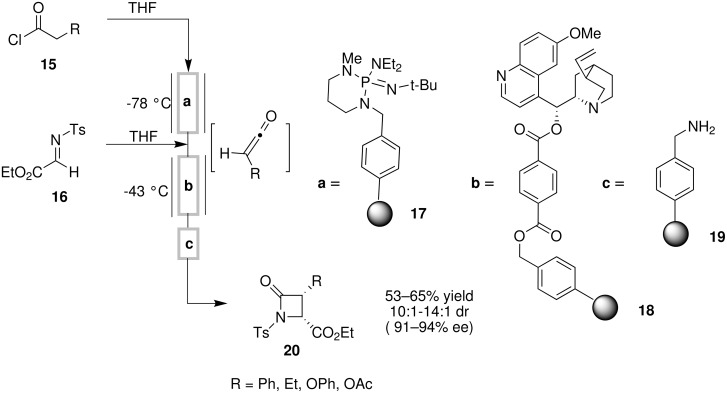
Asymmetric synthesis of ß-lactams.

Cinchona alkaloid derivatives have been featured in a number of solid support-based continuous flow asymmetric reactions. For example, a Wang-resin supported quinine derivative was developed for the reaction of imino esters with ketenes, leading to the stereoselective synthesis of ß-lactams [30]. Ketene was generated from the corresponding acid chloride with polymer-supported BEMP **17**, and was reacted with an imino ester in a reaction catalyzed by the resin-supported quinine derivative **18**. In this way, the handling and isolation of reactive ketene intermediates was directly avoided. A long and rigid linker to the resin was found to be ideal for obtaining good selectivities in this reaction. Nucleophilic scavenger **19** was used to remove excess reagents and byproducts. This three-step sequence was carried out in a single process using an assembly of jacketed glass columns with gravity driven flow-through over the course of 2 h (Scheme 6). The catalyst column was reused 60 times without any observed decrease in activity or enantioselectivity.

**Scheme 7:**
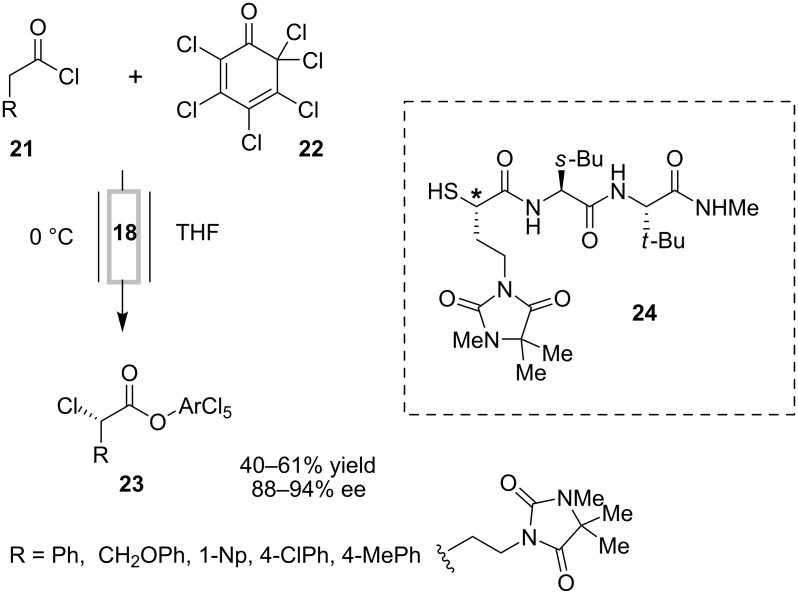
α-Chlorination of acid chlorides in flow.

Resin-immobilized quinine **18** was also employed in a similar flow system for the asymmetric α-chlorination of acid chlorides (Scheme 7) [31–32]. This cinchona alkaloid derivative served the dual purpose of dehydrohalogenation and asymmetric induction, and was found to be reusable at least up to 100 times, after regeneration each time by flushing with a solution of *i*-PrNEt_2_ in THF. A diastereoselective synthesis of the metalloproteinase inhibitor BMS-275291 **24** was developed using this column-based flow approach, with this α-chlorination reaction as a key step (Scheme 7) [32].

**Scheme 8:**
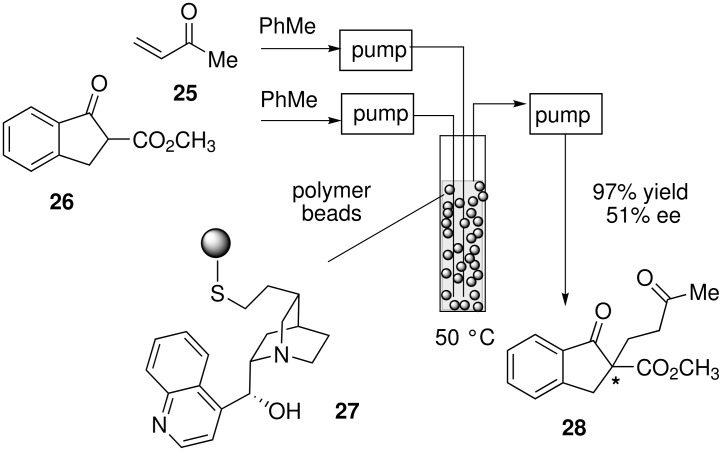
Asymmetric Michael reaction in continuous flow.

The Michael addition of indanone **26** to methyl vinyl ketone was investigated in flow using polystyrene-supported cinchonine and cinchonidine **27** (Scheme 8) [33]. A fluid bed reactor was devised for this asymmetric reaction, which allowed for the polymer beads to move around freely. Solutions of the reagents were introduced into the bottom of the column bed, and the reactants were removed from the top via peristaltic pumping. At a flow rate of 5.0 mL h^−1^ (residence time of ca. 6 h), a high yield of the Michael addition product **28** was obtained with enantioselectivity comparable to results obtained in batch with free cinchonidine.

Macroporous monolithic materials as solid-supports for catalysts and reagents have been found to be particularly well-suited for flow chemistry [34–36]. These rigid structures have large surface areas, leading to improved mass-transfer between the supported catalyst with the liquid phase and do not suffer from large pressure drops during flow-through that can often be problematic with gel-type resins. The flexibility and ease for adjusting porosity, composition and shape of these materials is an additional advantage [37–38].

**Scheme 9:**
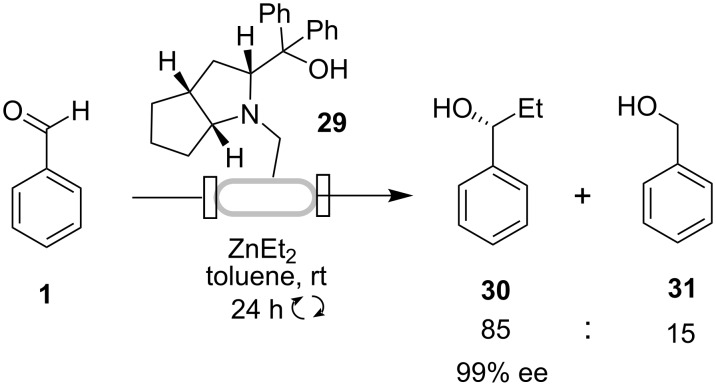
Enantioselective addition of Et_2_Zn to benzaldehyde using monolithic chiral amino alcohol.

The monolith-supported chiral amino alcohol catalyst **29** has been developed for the enantioselective addition of diethylzinc to benzaldehyde [26]. The monolithic catalyst was prepared as a column that was attached to a pump and a reservoir of the reagents. The reaction mixture of benzaldehyde and an excess of Et_2_Zn was then pumped and recirculated through the column over 24 h, resulting in complete conversion of benzaldehyde to the desired (*R*)-alcohol **30** with 85:15 selectivity and 99% ee (Scheme 9). Lower enantioselectivities were observed in batch reactions using a homogeneous analogue (87% ee) and also for a heterogeneous analogue with the catalyst grafted onto Merrifield resin (89% ee). The monolith-supported catalyst **29** also demonstrated potential long-term stability. It was reused four times (each a 24 h cycle) successively, with the fourth cycle being carried out 2 weeks after the first, without any changes in catalytic activity.

It should be noted that the support medium can have a profound effect on accessibility to the catalytic sites, creating microenvironments which can lead to quite different catalytic behavior compared to that observed in solution phase. Even the use of different polymeric supports can lead to significant perturbations in selectivity, as illustrated by an investigation on the use of Ti-TADDOL-functionalized monolithic columns for Diels-Alder cycloaddition of cyclopentadiene and 3-crotonyl-1,3-oxazolidin-2-one [39]. In this case, a complete reversal of topicity was observed, switching from a monolithic catalyst to the one grafted on a polymer matrix.

**Scheme 10:**
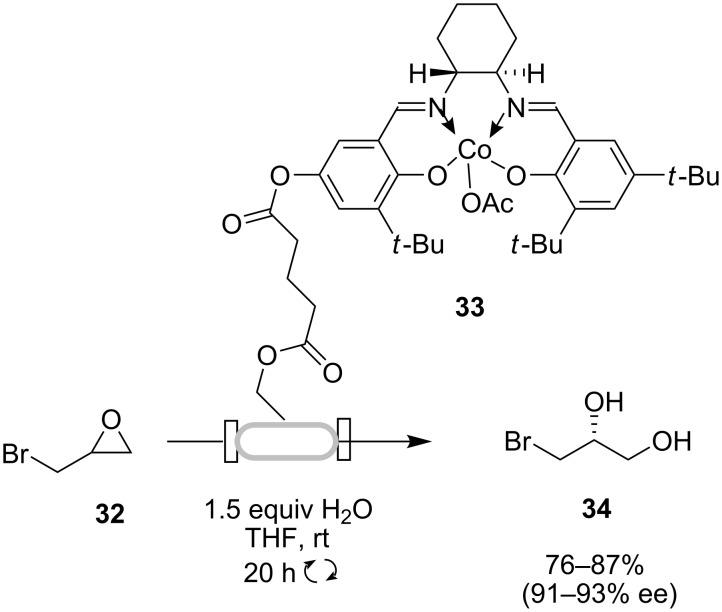
Continuous-flow hydrolytic dynamic kinetic resolution of epibromohydrin (**32**).

Kirschning and co-workers have designed a continuous-flow reactor system, PASSflow [40], based on the use of a functionalizable monolithic rod derived from a glass/polymer composite. This device was used for the dynamic kinetic resolution of epibromohydrin **32**, using a monolith reactor functionalized with a chiral Co(salen) complex **33** (Scheme 10) [41]. Three consecutive 1 mmol scale runs (20 h each), where a solution of epibromohydrin was continuously circulated through the reactor via a pump, were performed without any loss of enantioselectivity and catalyst activity (76–87% yield, 91–93% ee). A fourth, larger scale run (10 mmol) was performed continuously over a 6 day period (until complete consumption of epibromohydrin), providing the desired (*R*)-diol **34** also in similar yield and enantioselectivity. The hydrolytic kinetic resolution of a terminal epoxide in continuous flow has also been investigated using a silica-supported chiral Co(salen) complex [42].

**Scheme 11:**
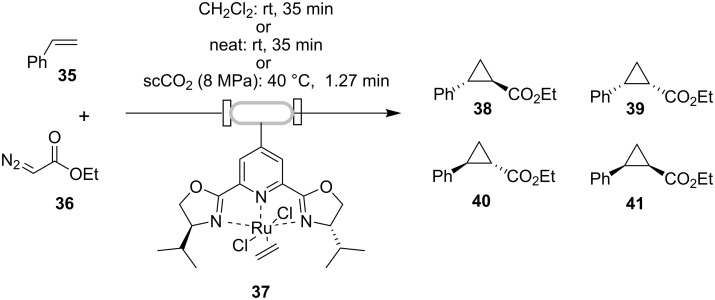
Continuous-flow asymmetric cyclopropanation.

Asymmetric cyclopropanation has also been studied in continuous flow, using monolithic reactors immobilized with chiral PyBox ligands [43–44]. The cyclopropanation of stryrene with ethyldiazoacetate was investigated as a model reaction for this asymmetric process (Scheme 11). The Ru-PyBox complexes were generated by flow-through of a solution of dichlororuthenium(II) (*p*-cymene), followed by washing to remove uncomplexed material [43].

The yields, trans/cis selectivity and enantioselectivities of runs performed in flow with the monolithic reactors were found to be comparable to the analogous reaction carried out in batch under homogeneous conditions. Multiple runs (each lasting 5–8 h) were performed in the reactors with consistent results during as well as between each run. These continuous flow reactions were first conducted in CH_2_Cl_2_ and neat conditions as well as in supercritical carbon dioxide (scCO_2_), as means of creating a more ‘environmentally-friendly’ process [45]. Under the solventless or scCO_2_ flow conditions, the productivity of the catalyst was observed to be much higher.

**Scheme 12:**
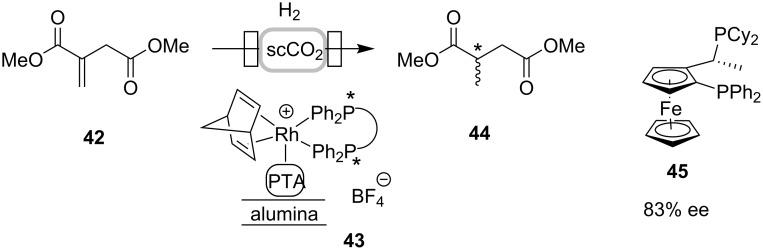
Continuous asymmetric hydrogenation of dimethyl itaconate in scCO_2_.

The use of supercritical fluids, in particular scCO_2_, as an alternative reaction medium to traditional organic solvents is attractive not only for environmental reasons, but also because of its high miscibility with gases, and its ease of removal from the product (simply by depressurization) [46]. In an early example of asymmetric hydrogenation in flow, ethyl pyruvate was reduced using a cinchonidine-modified Pt/Al_2_O_3_ catalyst in a fixed bed reactor in both supercritical carbon dioxide and ethane [47]. Poliakoff and co-workers have investigated the continuous asymmetric hydrogenation of dimethyl itaconate in scCO_2_ [48–49]. By using the catalyst [Rh(COD)_2_(nbd)]^+^[BF_4_]^−^, immobilized on alumina via a phosphotungstic (PTA) linker H_3_O_40_PW_12_, a variety of chiral bisphosphine ligands were screened for improving enantioselectivity [49]. The best enantioselectivity (83% ee) was obtained using Josiphos ferrocenyl ligand **45** at 55 °C and 16 MPa (Scheme 12).

**Scheme 13:**
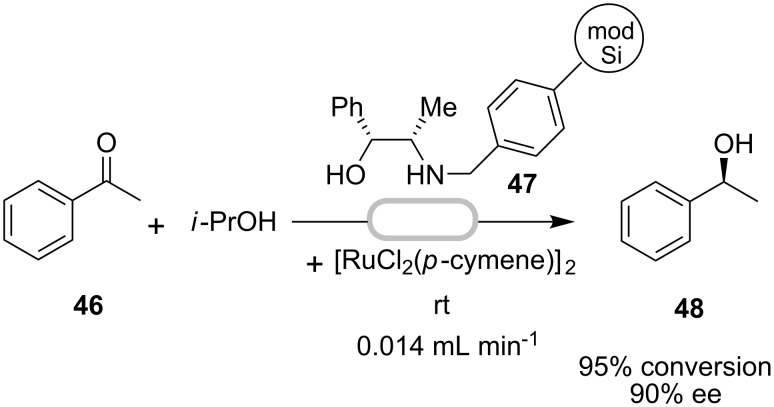
Continuous asymmetric transfer hydrogenation of acetophenone.

Silica has also been used as a heterogeneous support medium in continuous flow. A ruthenium catalyst complexed to a norephedrine-derived ligand **47** was immobilized onto modified (alkylsilyl-capped) silica and employed to catalyze a continuous asymmetric transfer hydrogenation [50] (Scheme 13).

The use of unmodified silica lowered the activity of the catalyst system, presumably via adsorption of some of the Ru(II) catalyst. Under optimal flow conditions, the transfer hydrogenation of acetophenone in isopropanol (using a flow-reactor consisting of a column packed with a slurry of the immobilized catalyst) provided a steady state conversion of 95% to alcohol **48** with 90% ee. Less than 1% of ruthenium leaching was observed over 11 h of flow with consistent catalyst activity and selectivity throughout the process. At slower flow rates, enantioselectivities were found to be lower due to the opportunity for equilibration during the longer residence times. Incidentally, this silica supported catalyst system was active up to at least three weeks compared to the homogeneous catalyst that was stable up to 20 h only. This property is perhaps a consequence of favourable active site isolation [50]. Several other examples of asymmetric transfer hydrogenation in flow have also been reported [51–52].

**Scheme 14:**
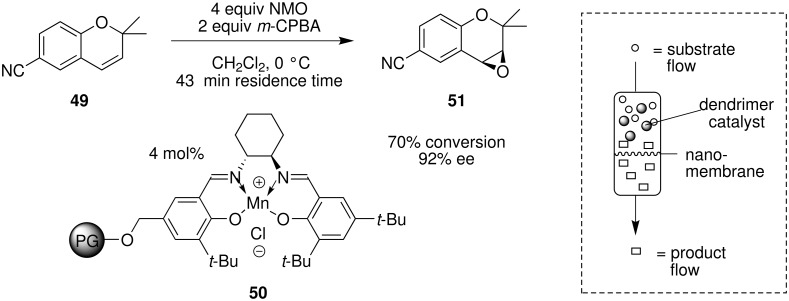
Asymmetric epoxidation using a continuous flow membrane reactor.

Supported catalysis has been extended to reactions involving the use of continuous flow membrane reactors [19–22]. For example, the asymmetric epoxidation of a chromene derivative **49**, catalyzed by homogeneous dendritic polyglycerol supported Mn-salen catalyst **50**, was recently developed in a continuous membrane flow reactor [53]. This type of flow system involves the continuous removal of the product from the high molecular weight macrostructured catalysts by filtration through a nanomembrane (Scheme 14). In this case, a stainless steel ultrafiltration cell was fitted with a solvent-stable MPF-50 nanomembrane that was able to retain up to 98% of soluble catalyst **50**. Under steady state conditions 70% conversion to the epoxide with up to 92% ee was achieved.

### Biocatalysis

**Scheme 15:**
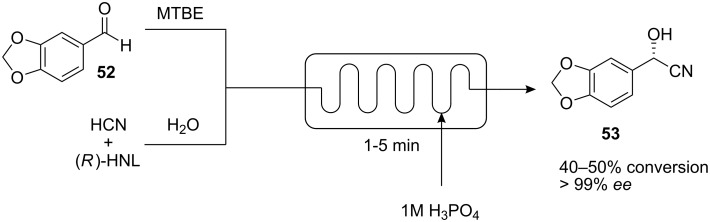
Enzymatic cyanohydrin formation in a microreactor.

The usefulness of enzyme-driven catalysis as a clean and efficient method for effecting chemical transformations has found applications in a number of industrial processes, some of which rely on continuous flow [54]. Recently, efforts have been made to combine the advantages of micro flow devices with enzymatic transformations. For example, Rutjes and co-workers have developed a method using a borosilicate microreactor chip for the enantioselective formation of cyanohydrins from aldehydes using hydroxynitrile lyase (HNL)-containing crude cell lysates (Scheme 15) [55]. The flow device consisted of three inlets: one for the aqueous phase delivering the cell lysate, KCN and citric acid (*in situ* HCN generation), one for the organic phase containing the aldehyde substrate and a third one at the end of the reactor channel as quenching inlet for deactivation of the enzyme. Under batch conditions, these reactions required vigorous stirring in order to maintain a constant emulsion that was necessary for high yields and enantioselectivities. Pillar-structured channels in the microreactor were designed to facilitate biphasic laminar flow. However, undefined plug flow was observed instead. Various aldehydes were tested using the system; with aromatic substrates, enantioselectivities higher than 95% ee were obtained and with an aliphatic substrate, 85% ee. Screening experiments were performed with piperonal **52** as the substrate, to evaluate the effect the ratio of the two phases and the reaction time have on the enantioselectivity and conversion (Scheme 15). The results were found to be similar to the batch results where steady emulsions were formed. Up to 58 reactions were performed serially within 4 h, requiring a total of only 150 μL of cell lysate.

**Scheme 16:**
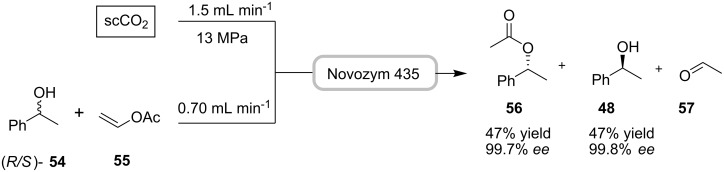
Resolution of (*R/S*)- **54** with immobilized lipase in a continuous scCO_2_- flow reactor.

Numerous applications involving the enzymatic resolution of racemic substrates in flow devices have been developed recently, including processes using alternative solvents such as ionic liquids and/or scCO_2_ [56–61]. The kinetic resolution of racemic 1-phenylethanol **54** was performed continuously in scCO_2_, using an immobilized lipase (*Candida antarctica*, Novozym 435) and vinyl acetate (**55**) (Scheme 16) [61]. This reaction in continuous flow proceeded to give both (*R*)-acetate **56** and in 99.7% ee, in 47% yield and the (*S*)-alcohol **48** in 99.8% ee, in 47% yield. In comparison to the reaction in a 10-mL batch reactor (0.83 mmol in 7 h), a throughput of 25 mmol h^−1^ was achieved in the 5-mL continuous flow reactor when 221 g of alcohol **48** were processed, providing consistent results over a period of 3 days.

**Scheme 17:**
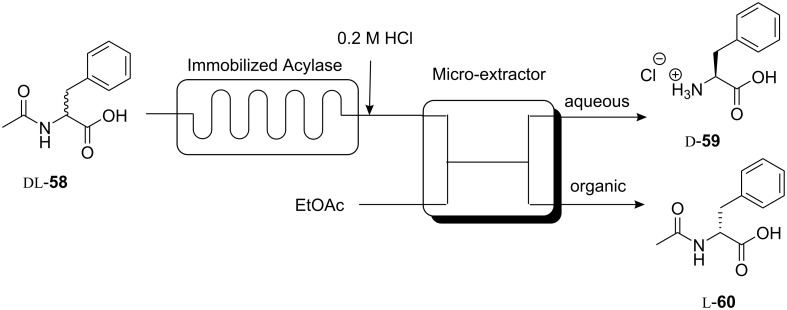
Enantioselective separation of Acetyl-D-Phe in a continuous flow reactor.

An interesting example of a chiral separation using a cross-linked polymeric acylase aggregate immobilized in a microreactor was reported by Maeda and coworkers [57]. Taking advantage of a continuous flow system, the resolution of a mixture of the racemic acetyl-amino acid (DL-**58**) was carried out in tandem with a microextractor for the selective separation of the resulting chiral products (Scheme 17). The racemic substrate was introduced into the microreactor in a buffered aqueous solution, then acidified with HCl subsequent to reaction completion, and combined with ethyl acetate organic phase for the extraction step. A chemically modified channel with hydrophobic/hydrophilic surfaces was used for the microextractor in order to maintain consistent laminar flow. Laminar flow is important for good interfacial mass transfer and efficient extraction. Under optimal flow rates for both the aqueous and organic phases, almost 100% of L-Phe **60** (99% ee) remained in the aqueous phase, while up to 84–92% of the acetyl D-amino acid **59** was extracted into the organic phase (Scheme 17).

## Conclusion

Asymmetric reactions have been carried out under both homogeneous and heterogeneous conditions in flow. Good control of reaction parameters is particularly important for these transformations, where minor changes in reaction parameters can have an adverse effect on stereoselectivity. Under flow conditions, reliable and consistent stereoselectivities as well as yields can be maintained; scale-up and rapid screening is also facilitated. While many notable examples of asymmetric synthesis using continuous flow technology have been reported, it is certain that many more applications will be developed in the near future.
